# New realities for Polish primary school informatics education affected by COVID-19

**DOI:** 10.1007/s10639-021-10778-8

**Published:** 2021-11-24

**Authors:** Taras Panskyi, Ewa Korzeniewska, Małgorzata Serwach, Krzysztof Grudzień

**Affiliations:** 1 Institute of Applied Computer Science, Lodz University of Technology, Stefanowskiego 18/22, 90-537 Lodz, Poland; 2 Institute of Electrical Engineering Systems, Lodz University of Technology, Lodz, Poland; 3 Faculty of Law and Administration, University of Lodz, Lodz, Poland

**Keywords:** Distance learning, Primary school, Information technology, Covid-19

## Abstract

In the paper, the authors discuss the first research effort to explore the transition from traditional teaching into distance teaching in Polish primary schools enforced by COVID-19. The first research question was addressed to primary school students and was dedicated to furnishing them with ICT equipment for crisis-prompted distance informatics learning. According to the obtained results, almost all Polish students during the pandemic have a technical opportunity to participate in distance learning and to use digital devices to develop their digital competences. Hence the second research question was addressed to the experts, demystifies whether the accessibility and the availability of ICTs could increase students’ informatics learning outcomes in out-of-school primary education settings. The obtained results reveal the significant importance of out-of-school informatics education in pandemic time. Moreover, in the first wave of pandemic, distance informatics education had the same or similar effect as if students learn informatics by themselves, without school lessons and teachers’ support. The obtained results should strengthen teachers and school leaders in making informed decisions during the shift into distance informatics education. Also, by investigating participants’ informatics learning outcomes and teacher preparedness and choices when implementing distance education, authors hope that the study may be helpful for policy-makers with the progressive changes in education and government support for informatics, especially in Poland, in making informed decisions to aid the transition into distance education as well as developing preparedness plans for future pandemics.

## Introduction

The World Health Organization announced a coronavirus pandemic on March 11, 2020. WHO General Director - Tedros Adhanom Ghebreyesus said that the SARS-CoV-2 coronavirus-caused COVID-19 disease is of a mass nature and poses a widespread public health threat worldwide. Following the position of WHO, a pandemic was declared in Poland. It is valid from March 20, 2020, until further notice. Comprehensive legal regulations relating to counteracting the COVID pandemic have been provided for in the provisions of the Act of March 2, 2020, on special solutions related to the prevention and combating of COVID-19, other infectious diseases and the emergencies caused by them (Kancelaria Sejmu, 2020). The government of the Republic of Poland did not decide to introduce a state of emergency, it successively introduced restrictions on social and economic life, which in the period of their greatest intensity were commonly referred to as “lockdown”. They included a ban on leaving the place of residence, limiting the possibility of performing specific economic or professional activities, changes in the field of health protection, and they also granted state authorities new powers and competencies of a ruling nature. Lockdown also meant the closure of schools and other educational institutions. It took place on March 12, 2020. To ensure the possibility of teaching and health safety of students, a decision was made to introduce, on March 25, 2020, a distance education system with the use of synchronous and asynchronous communication e-learning tools (CEDEFOP, 2020). In this way, a thorough change in the way of teaching was made for over 5 million students and 24.5 thousand students in educational establishments. As the Ministry of National Education emphasizes in a special report that Poland is one of the countries that reacted fastest to the threat of infection of students and teachers with the coronavirus (MEN, 2020).

Despite the introduction of subsequent stages of the economy “defrosting”, it was not decided to formally open schools and return to traditional methods of education. Thus, the distance teaching mode was conducted from March 25, 2020, until the end of the school year. In Poland that period has been referred to as the first wave of the COVID-19 pandemic. The restrictions were lifted only in relation to kindergartens (from May 6, 2020) and grades I - III of primary schools (from May 25, 2020). Distance education that was only an option a few months ago became a necessity. It is worth noting that distance education before the pandemic can only partially help us act meaningfully today. Before the pandemic, the students or their parents could decide whether to use distance education with its advantages (low geographical and financial barriers, flexibility and availability) or not. In the new conditions, we have a compulsion, which means that regardless of the possibilities, competencies and willingness, such solutions must be used by everyone, not only those who want and can. Moreover, the crisis-prompted primary school education reflects the shift from existed formal education both traditional and out-of-school to distance. This means that all forms of stationary education nowadays (during a pandemic) becomes distance only.

The education system in Poland is governed by Acts of Parliament and adopted ministerial regulations, in particular, by the Minister of National Education. A new reform in the primary school education system was initiated in the school year 2017/2018 and in high schools in the school year 2019/2020. Since 2017/2018, the school system at the primary level consists of the 8-year single structure primary education for students aged 7–15/16. Moreover, primary education is divided into two ISCED levels: early school education (grades I-III) and subject-based primary education (grades IV to VIII). With respect to the new reform, all children are required to learn informatics (in Poland “informatics” is equivalent to “computer science”) in primary schools (Sysło, 2019; Sysło & Kwiatkowska, 2015). With respect to the new Core Curriculum, in grades I-III, key skills to be developed by informatics include the use of linear order repeating actions (e.g. arranging patterns, pictures, events in a logical sequence); the use of technology resources (e.g., puzzles, logical thinking programs, digital cameras, drawing tools) for problem-solving; the use of the online resources (e.g., online discussions, Web environments) to participate in collaborative project-based activities; competencies such as creativity and innovativeness. Students know how to describe internal and external parts of computing devices; are able to organize and present collected data visually to highlight relationships and support a claim; could create simple programs in block-based visual environments that include sequences, events, loops, and conditionals. In grades IV-VIII, key skills to be developed by informatics contains a section on algorithmic thinking, problem-solving using computers, computational thinking, and game-based learning. Students could design projects that combine hardware and software components to collect data; model the role of protocols in transmitting data across networks; collect data using information and communication technology (ICT) tools and transform the data to make it more reliable, use flowcharts to address complex algorithms; design programs that combine control structures, including nested loops and compound conditionals, decompose problems into parts to facilitate the implementation of programs (Panskyi et al., 2019). Moreover, students develop critical and logical thinking, as well as reasoning, argumentation and deduction skills; creative solving of problems in various areas while using purposefully ICT-based methods and tools, including programming; solving problems with the use of mediation techniques.

The Polish school had to face many problems, doubts and issues that arose not only when conducting distance lessons, but also during the development and implementation of distance learning methods by teachers (Jaskulska & Jankowiak, 2020). When conducting remote classes in informatics, one of the main problems was the selection of appropriate tools enabling the effective implementation of the lessons (Mogalo, 2020). Due to the lack of clear guidelines regarding the choice of software and the method of conducting lessons, teachers used Google Classroom, Webex, or Moodle, for remote classes in an asynchronous manner or Zoom, MS Teams in synchronous teaching. Some teachers also tried to adapt remote lessons, both for students using computers and laptops and for students who only had the opportunity to participate via mobile devices and tablets (Dobosz, 2020). However, access to ICT devices was only one of the factors affecting the ability to establish remote communication between the teacher and the student during the informatics lessons.

The most common technique, used in the first wave of informatics distance education relied on emailing tasks to be done, chapters to read in textbooks or instructions to read from the Internet. Despite the fact, that informatics mostly did not require lab experiences, specialized equipment, materials, reagents or hands-on work as physics, chemistry or biology, some teachers omit informatics in favour of mathematics and language lessons. Generally, informatics lessons were conducted mainly during school hours, using traditional methods, with a small number of extracurricular activities. Nevertheless, the out-of-school activities remain an extension of the didactic and educational process of the school, accelerate the talented and gifted students, give them the opportunity to satisfy, develop and deepen their interest and creative work, introduce the pathways adapted to their individual needs in informatics-related fields. As a result, extracurricular activities that properly fulfil their intended functions may have a greater impact on students than crisis-prompted distance school informatics lessons.

The necessity of using ICT solutions could and should contribute to the growth of competences in the area of informatics. Therefore, the authors have made an attempt to investigate the impact of the first wave of pandemic period and the resulting limitations in distance education, as well as the need for using information technology on digital competences of primary school students. In this aspect, it became purposeful to pose two research questions:

- Do the students in primary school have access to the ICT devices which allow them to have distance learning via the Internet?
- Does the accessibility and the availability of ICTs could increase students’ informatics learning outcomes in out-of-school primary education settings during the crisis-prompted distance education?

## Methodology

### Design and procedure

Design for this study, a non-experimental research design, has been proposed, during the 2019/2020 academic year. Firstly, the research was conducted as an auditorium-internet survey. It is a variation of the CAWI (Computer Assisted Web Interviews) research technique, in which the research is carried out online using a questionnaire placed in a virtual educational environment. In a pandemic, when the limitations in direct contacts between people are high, an auditorium-internet survey is an appropriate tool to conduct research. The questionnaire was adapted to the age groups of primary school students. 10–14-year-olds answered the same questions, while the questionnaire for students aged 8–9 was prepared in the form of a picture, adapted to the reading skills and perception of students of this age.

For the second research question, the evaluation procedure was held during the pandemic COVID-19 period from March to June 2020. First, a descriptive analysis has been carried out followed by an inferential analysis. The level of significance established has been 0.05. In this study, a group of experts assessed the level of informatics skills acquired by Polish primary school students. Each expert evaluated the participants’ skills according to the criteria prescribed in the Polish National Informatics Curriculum.

### Participants

The study non-probabilistic sample consists of 333 primary school students from K3 to K10 curriculum standards in Poland. The following groups participated in the first research study:

- Students aged 8: 60 questionnaires were conducted;
- Students aged 9: 50 questionnaires were conducted;
- Students aged 10: 37 questionnaires were conducted;
- Students aged 11: 33 questionnaires were conducted;
- Students aged 12: 47 questionnaires were conducted;
- Students aged 13: 46 questionnaires were conducted;
- Students aged 14: 60 questionnaires were conducted.

The selection of students for the study was based on data from the nationwide Infosukces informatics contest (Panskyi & Korzeniewska, 2021). In each age group (except 9-year-olds), a slightly higher percentage of boys than girls have been examined (see Table 1).

**Table 1:** Participants of the survey by age and gender

Age	Gender	Quantitatively	Percentage (%)
8 years old	Boys	35	58.3
	Girls	25	41.7
9 years old	Boys	25	50.0
	Girls	25	50.0
10 years old	Boys	22	59.4
	Girls	15	40.6
11 years old	Boys	20	60.6
	Girls	13	39.4
12 years old	Boys	30	63.8
	Girls	17	36.2
13 years old	Boys	35	76.1
	Girls	11	23.9
14 years old	Boys	37	61.6
	Girls	23	38.4

To answer the first research question, the authors use a total sample of 333 students. In order to ensure that the results are not influenced by the size of the sample of students in comparison with the first research question, a random selection of students for the second research question has been made. 18% of the total sample (60 students) has been randomly selected and equally assigned to the group “A” – 20 students, “B” – 20 students and “C” – 20 students respectively. Group “A” corresponds to the students that have a school distance informatics education with additional distance out-of-school courses during the COVID-19 pandemic crisis. Group “B” corresponds to the students that have only school distance informatics lessons without any out-of-school activities. Group “C” corresponds to the students without any distance school informatics lessons and without out-of-school courses during schools’ lockdown. Group “C” reflects the failure in the crisis-prompted transition from traditional to distance informatics education when teachers universally perceive poor guidelines of the distance curriculum established standard requirements. As a result, teachers have not fully received support, with new textbooks accompanied by manuals with new pedagogic methodology, didactic tools, lesson plans, teaching aids (Plebańska et al. 2020), which caused a two-month (March–June) stagnation of informatics at primary school.

### Analysis of results

#### Question 1

In order to show the differences between the students’ answers, the same questions were asked to the same sample of 333 primary school students before the pandemic (October, 2019) and during the pandemic (March–June, 2020). It is important to distinguish what authors mean by ICT access and ICT use. ICT access refers to availability of ICT devises and services for use by any member of the household at any time, independently or jointly of whether the ICT device is owned or not by the household. Use of ICT refers to use by one or more members of the household, either at home or elsewhere.

**Fig. 1:**
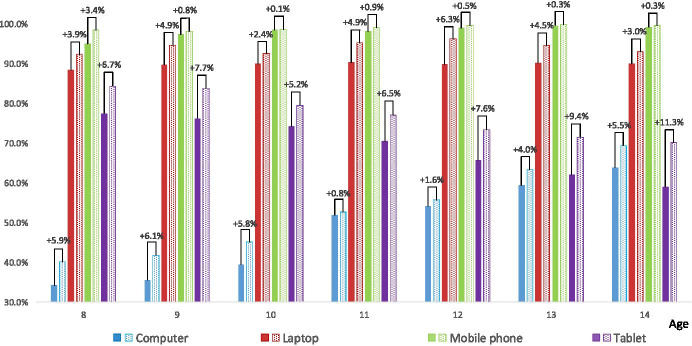
The access to the ICT devices by student households - Q1 (multiple answer question). The solid fill bars indicate the situation before the pandemic, the pattern fill bars – during the pandemic. The differences between students’ feedback towards the ICT devices available in their households, before and during the pandemic, are marked with a plus sign in favour of the second

In all age categories, the question marked as Q1: “which of the listed devices cover the access of your household ICT infrastructure” was asked. Almost every primary school student declares the access to mobile phones (over 98% of indications at all ages). Laptops were ranked as the second (over 92% of responses at all ages). The older the student, the more often he/she declares that his/her household access to the computer for common use. The opposite situation occurs in the case of the tablet - the older the student, the less frequently he/she declares that his/her household has access to tablets. Figure 1 also shows the access to the ICT devices by students’ household before the pandemic came. The overall pattern remains the same before and during the pandemic, with a slight upward trend in the 2020 year. The overall differences were indicated in access to all ICT devices at all ages in favour of the 2020 pandemic year. The biggest differences towards the access to computers have been indicated among 8–10-years-olds (5.8–6.1%). With respect to laptops, the biggest difference has been indicated among 12-years-olds (6.3%). The situation with mobile phones remains the same before and during the pandemic. The household’s access to tablets with its facilities and services shows a significant increase compared to all the previous ICT devices. The biggest difference has been indicated among the 14-years-olds (11.3%) and the smallest among the 10-years-olds (5.2%). The results show that one strong conclusion can be drawn: “thanks” to the pandemic students’ households have the increased available access to ICT infrastructure that could and definitely should significantly improve the quality of education.

Before the pandemic, in 2011 almost 67% of Polish households had at least one computer (Pekasiewicz & Szczukocka, 2017). According to the previous GUS (in English: Central Statistical Office) study (GUS, 2017), households showed positive dynamics of changes towards access to computers from 75% in 2013 to 82% in 2018. Their recent report (GUS, 2019) revealed that in 2019 almost 84% of Polish households had at least one computer. The presented results confirmed the finding described in the study (Librus, 2021) provided during the pandemic in 2020, which indicated that almost 92% of students had the access to computers and 72% to mobile phones. According to (Ostrowska & Sitek, 2020), less than 4% of students declared a lack of their access to a computer or tablet at home. These are probably the students with disabilities or living in extreme poverty (Federacja Konsumentów, 2021). Even though, the problem of digital exclusion was strongly emphasised by many teachers and parents during the first wave of the pandemic (Marchlik et al., 2021).

**Fig. 2:**
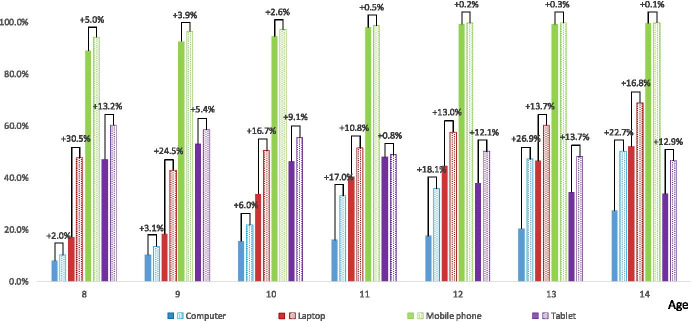
The ICT devices that belong to primary school students – Q2 (multiple answer question). The solid fill bars indicate the situation before the pandemic, the pattern fill bars – during the pandemic. The differences between students’ feedback towards the ICT devices they own, before and during the pandemic, are marked with a plus sign in favour of the second

In addition to asking Q1, primary school students were asked question marked as Q2: “which of the listed ICT devices they use belong only to them”, e.g. the parents bought them as a gift. Over 94% of the respondents during the pandemic period with the distance education learning mode indicated a mobile phone (see Fig. 2). In the case of computers, we can see the dependence that the older the student, the more often he/she declares that a computer was given/bought for their own use. The situation is completely different with tablets - it is the most popular ICT device the 8–10-year-old primary school students own, and the older the student, the less often he/she indicated that a tablet was given/bought for their own use. The laptop is the most popular among 13–14-year-olds.

Figure 2 also shows the differences between ICT devices that primary school students own and use before and during the pandemic. The biggest differences dedicated to the computers have been indicated among 13–14-years-olds (26.9–22.7%). With respect to laptops, the biggest differences have been indicated among 8–9-years-olds (30.5–24.5%). The crisis-prompted distance education and prolonged online learning has forced the parents/guardians to buy/get computers and/or laptops. Interestingly, for 8–9-years-old students, the tablets did not cope with their tasks as well as laptops and therefore the last ones aimed to overcome the technical, organisational and educational problems. With respect to tablets, the biggest difference has been indicated among 13–14-years-olds (13.7–12.9%). Tablets were not previously considered by adolescents as ICT devices appropriate for educational purposes, during the pandemic they became an alternative to laptops and computers. The situation with mobile phones remains stable before and during the pandemic.

According to (Ostrowska & Sitek, 2020) with respect to the PISA 2018 study, almost 28% of large families had only two computers, two tablets, or a computer and a tablet, and 8% had only one ICT device. Moreover, in 2019 almost 84% of Polish primary school students had their own mobile phones (UKE, 2019). During the first wave of the pandemic, this was already 94%. Tablets and smartphones are the handheld personal ICTs with convenient computing, communication and entertainment options, making them the most popular ICT devices to own (Pratama & Scarlatos, 2020). However, mobile phone or tablet ownership is less adequate to tell whether a student is digitally literate or not (Pratama & Firmansyah, 2021).

**Fig. 3:**
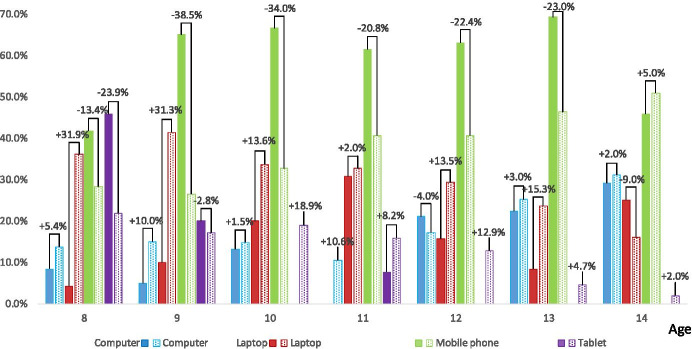
The ICT device the students used most often – Q3 (single answer question). The solid fill bars indicate the situation before the pandemic, the pattern fill bars – during the pandemic. The differences between students’ feedback towards the ICT devices before and during the pandemic are marked with a plus sign (in favour of the situation during the pandemic) and a minus sign (in favour of the situation before the pandemic)

From among the indicated ICT devices, students were to choose the one they used most often before and during the pandemic (question Q3). Figure 3 shows that before and during the pandemic the most popular ICT device was the mobile phone. It is worth adding that before the pandemic within the group of 9–13-year-olds, mobile phones were most often used by over 65%, 14-year-olds – by 45.8%, and 8-year-olds by 41.7%. Computers and laptops usage rates increase with the age. The opposite situation occurs in the case of tablets - the older the student, the less often he/she indicated this ICT device as the most frequently used. Moreover, among the group of 12–14-year-olds, before the pandemic, tablets were the least popular ICT devices. It is also worth adding that the most frequent use of tablets in the group of 8–9-year-olds were indicated by the youngest respondents: 45.8%.

The use of computers during the pandemic significantly increased. The biggest differences towards the usage of the computer have been indicated among 9 and 11-years-olds (10.0–10.6%). With respect to laptops, the biggest differences have been indicated among 8–9-years-olds (30.5–24.5%). However, the 12-years-olds use computers less frequently during the pandemic than before it came (−4.0%). Due to the crisis-prompted distance education use of laptops drastically increased during the pandemic. The biggest differences towards the usage of the laptop have been noticed among 8-years-old (31.9%) and 9-years-old (31.3%) students. However, with increasing age, the differences gradually decrease. With respect to mobile phones, during the pandemic, students reduced the use of this ICT device by almost twice. Students were overloaded with various virtual activities (Ptaszek et al. 2020) and the amount of time they were spending with new educational tools using ICT devices (generally laptops or tablets). Therefore, the use of these ICT devices predominate and mobile phones receded into the background. Nevertheless, mobile phones kept the leading position even during the pandemic. Tablets remain relatively affordable, which makes them relatively accessible to low income families. Therefore, during the pandemic challenging period, this type of ICT device became and alternative among 10–11-years-olds. However, the differences among using ICT devices among 8-years-olds indicate choosing more laptops (31.9%) and mobile phones (13.4%) than tablets (−23.9%).

**Fig. 4:**
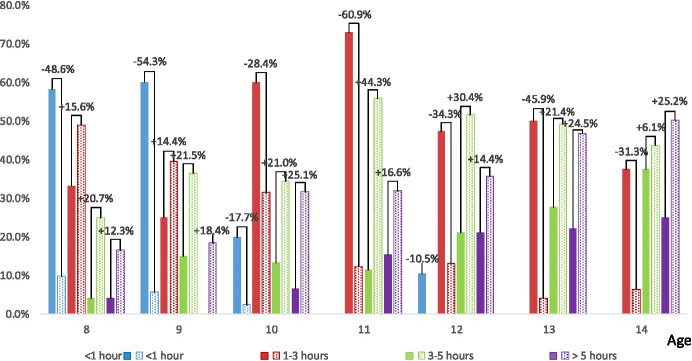
The amount of time (hours) the students spend using the ICT device they used the most – Q4 (single answer question). The solid fill bars indicate the situation before the pandemic, the pattern fill bars – during the pandemic. The differences between students’ feedback towards the time spend using the ICT device they used the most before and during the pandemic are marked with a plus sign (in favour of the situation during the pandemic) and a minus sign (in favour of the situation before the pandemic)

Figure 4 presents the time of using the most frequently used ICT device (most often a mobile phone) before and after the pandemic (question Q4). It shows that the older the student, the longer he/she use the device. In the group of 8–9-year-olds, before the pandemic, more than half indicated using the device for up to an hour (the highest percentage of 9-year-olds - 60.0%), and over 25% indicated up to 3 h a day (8-year-olds - 33.3%). In the group of 10–12-year-olds, the greatest percentage of older students from this group indicated the longer-term use of the device - from 3 to 5 h - 21.1% and over 5 h - 21.1% of 12-year-olds. In the group of 13–14-year-olds, 37.5% of the surveyed 14-year-olds indicated daily use of their favourite electronic device for more than 3 h a day, while 64.6% of 14-year-olds indicated the use of the device for more than 5 h.

The biggest differences in time spend using the ICT device is noticed in 8–9 years-olds. These students during the pandemic spent up to 1 h using their ICT device that is significantly less than before the pandemic came (−48.6% – 8 years-olds, −54.3% – 9 years-olds). Moreover, 11–14 years-old students, during the pandemic, did not use their ICT device less than an hour a day. The biggest differences in the use of the ICT device between 1 and 3 h a day is indicated in 11 years-olds (−60.9%). The group of 8–9 years-olds spend more time (1–3 h) during the pandemic than before. However, in the group of 10–14 years-olds the situation is the opposite. With respect to the time (3–5 h) the biggest differences are indicated among 11–12 years olds (44.3% – 11 years-olds, 30.4% – 12 years-olds). The smallest difference in this time interval is indicated among 14 years-olds (6.1%). Finally, the significant differences in the longest time interval (more than 5 h) occurred in 10 years-olds (25.1%) and 14 years-olds (25.2%). Moreover, all the students despite age spend more time (more than 5 h) using their ICT devices during the pandemic than before.

According to (CBOS, 2018) students aged 14–16 use their ICT devices approximately 4–5 h a day. Students aged 12 to 13 spent 4–5 h a day. The youngest (9–11 years old) use the ICT devices the least - from 2 to 3 h a day. A recent study (NASK, 2019a) revealed that before the pandemic in 2019, almost 15.3% of students spent 1–2 h using their favourite ICT, 27.3% spent 2–4 h and 22.9% spent 4–6 h. During the pandmic, in February 2020, the average time spent by adolescents using their ICTs was 5 h a day, in May 2020 this average jumped to 9 h a day (FEZiP, 2021). Hence, due to the pandemic, the crisis-prompted distance learning (including school and out-of-school activities) had forced primary school students to spend significantly more time using their ICT devices.

**Fig. 5:**
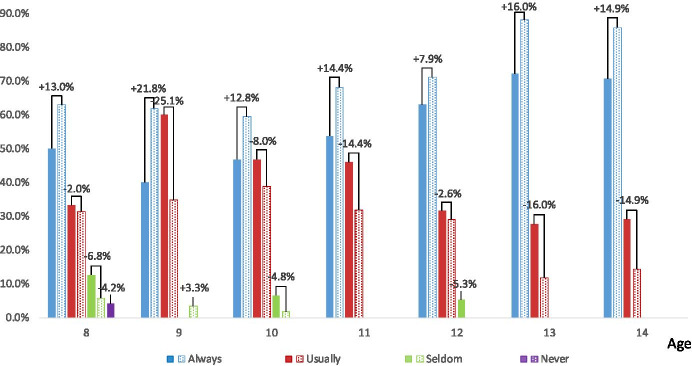
How often the students used the ICT devices to connect to the Internet – Q5 (single answer question). The solid fill bars indicate the situation before the pandemic, the pattern fill bars – during the pandemic. The differences between students’ feedback towards the frequency of using the most favourable ICT device before and during the pandemic are marked with a plus sign (in favour of the situation during the pandemic) and a minus sign (in favour of the situation before the pandemic)

Asking primary school students about the ICT devices they used the most often before the pandemic, the use of the Internet was not taken into account. Therefore, the question Q5 reveals how often students use their ICT devices in combination with an Internet connection. In the case of 8–9-year-olds, more than half of them declared that before the pandemic they always linked their favourite ICT device with an Internet. This percentage increases with age and from the age of 12–14, or more precisely from the age of 14, two-thirds of students declared that whenever they used an ICT device, they also used the Internet. Taking into account the sum of the answers: “yes – always” and “yes – usually”, the simultaneous use of ICT devices and the Internet is declared by over 88.3% of students aged 8–14. Only 4.2% of 8-year-old students used ICT devices without an Internet connection (see Fig. 5).

Due to the pandemic, the crisis-prompted distance education has forced students to study and to communicate only via the Internet. Despite the age, students always or usually have their ICT devices connected with the Internet. Therefore, almost all of the students had the opportunity to study in a distance learning mode. Nevertheless, the authors did not examine the quality of Internet connection and therefore we should be cautious in the interpretation of these findings.

According to GUS study (GUS, 2020b) in 2020, 89.6% of all households in Poland had broadband Internet access, which means an annual increase of this indicator by 6.3%, and compared to 2016 - by 13.9%. Another GUS research (GUS, 2020a) revealed that more than 91% of households with children had broadband Internet connection. Nevertheless, the resents research (Centrum cyfrowe, 2020) revealed that during the first wave of pandemic almost 38% of teachers identify the students’ problem with the Internet connection as the main problem in distance education. Moreover, almost 20% of teachers declared their problem with the Internet connection as the main issue during the second wave of pandemic (Buchner & Wierzbicka, 2020).

Reliable data from school-based online survey provides the quality ICT use data required to better inform education policy and practices, especially in Poland during the pandemic era. Capturing the complex set of factors involved will paint a more accurate picture of what is available and used by primary school students. This includes information, such as availability of digital infrastructure; internet connection speed; strategies implemented by schools to develop digital skills; and perceptions by principals and teachers on ICT use in education and its barriers. The use of ICTs for education during the COVID-19 crisis is a reality for which teachers and students must be better prepared. Nevertheless, the research reveals that primary school students in Poland are technologically prepared for the transition to distance learning, which is in line with the assumptions of the Ministry of National Education (MEN, 2020) and is also confirmed by the Report of Organisation for Economic Cooperation and Development (OECD, 2020a).

In this respect, the Polish primary school students during the pandemic reveal a high level of ICT access and ICT utilisation which is also associated with their better educational outcomes. In the past few years where access to ICT and the Internet have climbed worldwide, the focus has also shifted from the gap in access to ICT devices (Gurbiel et al., 2005) to the gap in ICT skills and digital literacy (Van Deursen & van Dijk, 2019). Digital literacy is not only associated with the access and the use of ICTs but also includes the Internet (Ferrari et al., 2012) and computer literacy (Webb et al., 2017; Webb et al., 2017; Webb et al., 2018), ICT proficiency and fluency. Moreover, digital literacy reflects the evolution, and give more weight to data literacy, media literacy and critical thinking (OECD, 2021a; Cirus & Simonova, 2021). The relations between ICT literacy, informatics and computational thinking is carefully described in (Sysło, 2019). According to (Sysło, 2020) the informatics goes beyond the use of ICT devices or applications since it helps to develop programming skills, logical and algorithmic thinking, creativity, computational thinking, and a systematic approach to problem-solving. Bracketing literacy–technology relations as networked, sociocultural, and multimodal, is focused on how ICT technologies fundamentally change the relationships of digital literacy to social relations (Downes & Brosseuk, 2021; Leander et al., 2015). Moreover, it is important to understand digital literacy as a form of social practice and to investigate the ways in which it interacts with ideologies and institutions to shape and define the possibilities and life paths of today’s ICT generation of students.

#### Question 2

Compulsory informatics education begins at age 6/7 and throughout primary education and later in secondary school, students acquire knowledge using a spiral learning model by building on foundational concepts with progressively deeper content coverage (Sysło, 2019). Systematically topics are revisited with increasing complexity that extrapolates previously presented information using the spiral learning model. According to this model, skills and knowledge verified during the maturity examination are developed by students from the very beginning of their education. To obtain the maturity certificate, students must acquire and use the knowledge passed on to them by their teachers not only during their secondary education, but also at primary school. For this reason, the authors of the study base their conclusions by analysing the basic scope of the maturity examination requirements according to the Polish National informatics curriculum. These issues should be mastered by students at the stage of their primary school education.

Maturity exam (matura) is the major form of external examination to ensure the comparability of learning outcomes. Every secondary school student who plans to study at the university must pass three mandatory Matura examinations. Matura is taken at the end of secondary education, by young people aged 18/19 years to enter the institutions of higher education. Moreover, the informatics Matura examination could not be chosen as a mandatory examination, so secondary school students took it as an additional examination at an advanced level. As a result, nowadays in Poland, there are no external standardized tests, exams or criteria that try to measure learning outcomes in primary school settings in the distance informatics domain. Moreover, there is no measuring tool to assess the success of distance informatics education.

Therefore, authors created/adopted a set of criteria that are closely related to the learning outcomes that primary students must comprehend in informatics via traditional face-to-face education according to the Polish National informatics curriculum. Thereafter, we imposed these criteria on the learning outcomes obtained by the primary school students with distance education and compared how close the students were to students without any informatics lessons in their knowledge acquisition, mastery of informatics skills, or development of their key abilities (Panskyi & Rowińska, 2021). Learning outcome requirements are the same in both face-to-face and distance education.

This set of criteria is based on an outcomes-based approach that makes our expectations more transparent to both the primary school students with distance education and without it. This approach starts with a specification of what the primary student will be expected to achieve during the long crisis-prompted period of transition into distance education. Moreover, the authors are interested if additional distance out-of-school extra-curriculum informatics activities could accelerate intellectual students’ development, thereby confirming or refuting the feasibility of such out-of-school activities as a bridge between traditional and distance school education (Panskyi et al., 2019). A set of criteria form the basis for the assessment framework including quality assurance checks and benchmarking for the validation process.

The validation set consists of 5 core criteria and their short names (see Table 2), where each covers the students’ learning outcomes of informatics at primary school according to the Polish National informatics curriculum. The academic teachers/experts could evaluate the students’ distance learning informatics outcomes by a set of criteria and mark one of four options in Likert’s scale, ranging from “Certainly achieve” to “Certainly not achieve”. They build their evaluation based on the systematic lesson observations. Before the pandemic, the observation practice took place in school placements, while during the lockdown, the practice has been transferred to virtual communication frameworks. The instrument is made up of five criteria or dimensions: understanding, analysing and problem-solving (CR1); programming and troubleshooting using computers and other ICT devices (CR2); the use of computers, ICT devices and computer networks (CR3); developing social competences (CR4); observing the law and safety rules (CR5).

**Table 2:** A set of validation criteria towards the learning informatics outcomes

Item/Criterion	Short name
Understanding, analysing and problems solving: plans the next steps in problem-solving, including the basic stages of computational thinking (problem definition, the definition of models and concepts, finding a solution, programming and testing the solution); compares the operation of various algorithms for a selected problem, analyses algorithms based on their ready-made implementations; uses algorithms learned in primary school to solve problems in various fields; checks the correctness of the algorithms for sample data.	CR1
Programming and troubleshooting using a computer and other ICT devices: designs and programs solutions to problems in various fields, uses: input/output instructions, arithmetic and logic expressions, conditional instructions, iterative instructions, functions with and without parameters, tests the correctness of programs for various data; correctly selects IT environments, applications and resources to implement solutions to problems, also uses elements of robotics; prepares solutions to problems using selected applications: creates extensive presentations, creates a website in accordance with standards, develops documents on various topics, collects data from various sources in a spreadsheet table; searches the web for the necessary information and resources, evaluates their usefulness and uses them to solve problems.	CR2
Use of computer, digital devices and networks: learns about the possibilities of new digital devices and accompanying software; explains the functions of digital devices other than a computer and uses their capabilities; solves problems using different operating systems; characterizes the Internet network, its general structure and services, describes the basic topologies of a computer network, presents and compares the principles of operation and functioning of a client-server and peer-to-peer computer network, describes methods of identifying computers in the network.	CR3
Developing social competences: actively participates in the implementation of IT projects which solve problems in various fields, taking various roles in the project implementation team and presenting the effects of teamwork; gives examples of the influence of informatics and computer technology on the most important spheres of personal and professional life; uses selected e-services; describes the impact of technology on the welfare of societies and social communication; explains the consequences of exclusion and the positive aspects of digital inclusion; presents the benefits of information technology and computer technology for people with special needs; broadens and complements its knowledge using the resources available on e-learning platforms.	CR4
Compliance with the law and safety rules: complies with the principles of netiquette and legal regulations regarding: personal data protection, information protection, as well as copyright and intellectual property protection in access to information; is aware of the consequences of breaking these rules; respects the applicable law and ethical standards regarding the use and distribution of computer software, third party and own applications and electronic documents; uses good practices in the field of protection of sensitive information (e.g. passwords, PINs), data and operating system security, explains the role of information encryption; describes the damage that piracy activities on the Internet can cause to individuals, selected institutions and society as a whole.	CR5

In order to verify the reliability of the instrument in this research, the Cronbach’s alpha test has been used. Analysis of internal consistency showed that Cronbach’s alpha was 0.76 for a set of 5 criteria, indicating good scale reliability. In addition, an exploratory factor analysis (EFA) was carried out using the main components analysis with varimax rotation. The results obtained with the sample adequacy index Kaiser-Meyer-Olkin (KMO) were 0.80 and Bartlet’s test of sphericity was significant (sig = 0.000), indicating that the correlation matrix exceeded the conditions for carrying out validation analysis.

### Descriptive and comparative analysis of each dimension of the instrument

In Table 3, each dimension of the instrument between groups “A”, “B”, “C” of students is analysed. The description of the groups is mentioned above in Section 2.2. This table includes the mean score and standard deviation of five experts (see Appendix). Regarding attitudes towards the CR, the authors could highlight some interesting results. The overall mean score indicates rather low level of informatics learning outcomes, between “Rather not achieve” and “Certainly not achieve”. However, students with distance informatics education with extra curriculum distance courses achieve a much higher mean score (M = 2.98), when students only with distance education and without it have a similar average (M = 3.45) and (M = 3.51) respectively.

**Table 3:** The descriptive statistics of the analysed dimensions of the instrument regarding groups

Dimension/Criterion	Group name	Mean	Std. Deviation
CR1	A	2.98	0.21
	B	3.45	0.16
	C	3.51	0.20
CR2	A	1.98	0.22
	B	2.67	0.17
	C	2.76	0.24
CR3	A	2.78	0.29
	B	3.08	0.35
	C	3.06	0.21
CR4	A	1.55	0.23
	B	1.68	0.19
	C	1.80	0.26
CR5	A	3.02	0.15
	B	3.05	0.18
	C	3.07	0.19

Regarding the CR2 the highest mean show students with extra distance courses in conjunction with school distance informatics (M = 1.98), while the mean of groups “B” and “C” practically do not differ (M = 2.67) and (M = 2.76) respectively. In general, the additional out-of-school distance informatics courses have a tremendous positive impact on the achievement of the CR2. Correctly incorporated informatics courses in distance education help students to enhance their skills and competences.

Similar results are found with respect to the CR3, however, additional distance informatics courses do not have such a strong impact here (M = 2.78). This criterion indicates the learning outcomes towards the ICT, internet and computer network. Among the results to highlight, it is observed that despite the students’ perception and attitudes towards new technology the informatics learning outcomes remain very low.

According to Berge (1995), the teachers should use techniques and approaches to create a friendly and social environment, organize and manage the interactions, make students comfortable with the system/software they are using in distance education. The CR4 shows the extremely high mean scores (M = 1.55; M = 1.68; M = 1.80) for group “A”, “B” and “C” respectively. Based on the data analysis, the research findings indicated that “social competencies” are the most developed in distance information education.

Regarding the last CR5 indicating the “compliance with the law and safety rules”, all three groups (M = 3.02; M = 3.05; M = 3.07) show the relatively low students’ knowledge towards e-safety or online security. Thus, the understanding of protection/safety measures, the use of strong passwords, safeguards, encryption procedures and data security remains unsatisfactory. Interestingly, the lack of distance informatics education has no effect on the deterioration of CR5 learning outcomes. On the other hand, the additional out-of-school distance informatics activities do not lead to greater students’ perception of personal data and privacy in digital environments.

### Anova analysis according to the different groups and dimensions

This section has analysed whether there are statistically significant differences regarding the informatics learning outcomes based on the five criteria of the instrument, based on the different established groups. Analyses of each dimension in relation to each of the sample groups are presented below.

#### CR1 dimension towards the students’ informatics learning outcomes

To verify the significance of the model proposed in relation to the different groups, a one-way ANOVA has been used, specifically by multiple comparisons. Levene’s homoscedasticity assumption is fulfilled F(2,57) = 1.06, p. > 0.05, so multiple comparisons have been made by Sidak. ANOVA determined that the proposed model was significant in inter-group variable F(2, 57) = 47.13, p. <0.05.

Partial eta-squared was calculated to determine the size of the effect (see Table 4). According to Richardson (2011), the partial eta square suggested values of approximately 0.01, 0.06 and 0.14 indicate small, medium and large effects, respectively. In this dimension, a large effect has been found (*η*^2^ = 0.623). As seen in Table 4, there are statistically significant differences in CR1 between group “A” and group “B” (p. = 0.000), as well as between group “C” and group “A” (p. = 0.000). That differences show how informatics oriented extra-curriculum distance courses could fundamentally boost the student CR1 learning outcomes. Nonetheless, there were no differences between group “B” and group “C” (p. = 0.705), which shows how imperfect and poor school crisis-prompt distance education is in building and managing the teaching informatics solutions.

**Table 4:** Multiple comparisons of CR1 towards the students’ informatics learning outcomes

(I) Group	(J) Group	Mean Difference (I-J)	Std. Error	Sig.	95% Confidence Interval	
					Lower Bound	Upper Bound
A	B	−0.473	0.060	0.000	−0.620	−0.325
B	C	−0.058	0.060	0.705	−0.206	0.089
C	A	0.531	0.060	0.000	0.383	0.679

#### CR2 dimension towards the students’ informatics learning outcomes

For the CR2 dimension, Levene’s homoscedasticity assumption is fulfilled, F(2,57) = 2.683, p. > 0.05, so multiple comparisons have been made by Bonferroni. ANOVA revealed that the proposed model was significant in the inter-group variable, F(2,57) = 82.081, p. <0.05. Partial eta-squared indicated large effect size (*η*^2^ = 0.742).

Similar results are found in CR2. Table 5 shows that multiple comparisons revealed that there were significant differences in group “A” and group “B” (p. = 0.000), as well as between group “C” and group “A” (p. = 0.000). However, there were no differences between group “B” and group “C” (p. = 0.498). Most of the Polish out-of-school distance informatics courses focus on the visual-based or text-based programming languages that enrich and enliven CR2 student learning outcomes. School distance informatics have produced questionable results in primary education, and this presumably reflects the fact that teachers were not prepared for such a development.

**Table 5:** Multiple comparisons of CR2 towards the students’ informatics learning outcomes

(I) Group	(J) Group	Mean Difference (I-J)	Std. Error	Sig.	95% Confidence Interval	
					Lower Bound	Upper Bound
A	B	−0.688	0.066	0.000	−0.852	−0.523
B	C	−0.093	0.066	0.498	−0.257	0.070
C	A	0.781	0.066	0.000	0.617	0.945

#### CR3 dimension towards the students’ informatics learning outcomes

As in the CR2 dimension, Levene’s homoscedasticity assumption is fulfilled, F(2,57) = 0.815, p. > 0.05, therefore, the multiple comparisons have been made by Tukey. The ANOVA analysis justified the significant differences between the analysed groups in the proposed model, F(2, 57) = 10.442, p. < 0.05, with a large effect size (*η*^2^ = 0.268).

Table 6 shows the result that follows a similar pattern to that in CR1 and CR2. There are substantial differences in CR3 between group “A” and group “B” (p. = 0.000), as well as between group “C” and group “A” (p. =0.005). However, no significant differences between group “B” and group “C” have been noticed (p. = 0.533). The CR3 corresponds generally to the ICT and computer networks. Due to poor development of infrastructure and a lack of equipment and skilled personnel in polish primary schools, the ICT penetration to school education has remained low. Moreover, the computer network issues in primary school (data communication, network models and protocol) are usually postponed by teachers to secondary or even get straight departure to higher education. Therefore, the analysis reveals no significate differences in CR3 with or without distance school informatics education.

**Table 6:** Multiple comparisons of CR3 towards the students’ informatics learning outcomes

(I) Group	(J) Group	Mean Difference (I-J)	Std. Error	Sig.	95% Confidence Interval	
					Lower Bound	Upper Bound
A	B	−0.371	0.084	0.000	−0.574	−0.167
B	C	0.091	0.084	0.533	−0.112	0.294
C	A	0.280	0.084	0.005	0.076	0.483

#### CR4 dimension towards the students’ informatics learning outcomes

Multiple comparisons for the CR4 dimension have been made by Bonferroni due to Levene’s homoscedasticity assumption, F(2, 57) = 0.995, p. > 0.05. The proposed model with differences determined between the analysed groups has been significant, F(2, 57) = 16.218, p. < 0.05, with a large effect size (*η*^2^ = 0.363).

Table 7 shows the significant differences in CR4 between group “C” and group “A” (p. = 0.000), as well as between group “B” and group “C” (p. =0.002). However, no significant differences between group “A” and group “B” have been noticed (p. = 0.149). The CR4 is responsible for the developing of students’ social competences including the interactions between coevals in school and students-teacher in the classroom. Distance informatics learning during the pandemic has hit vulnerable students the hardest. Despite the low-quality of Polish distance education, the results show that teacher’ efforts to fill this gap are yielding partial positive results. Given the human supports required by many primary students in order to flourish socially, emotionally and academically, it’s a challenge for the Polish educational system to provide the right types and amounts of distance informatics learning opportunities and supports. Therefore, group “A” and group “B” have not significant differences in CR4 learning outcomes. However, the absence of any informatics education – group “C”, could have far-reaching negative social consequences.

**Table 7:** Multiple comparisons of CR4 towards the students’ informatics learning outcomes

(I) Group	(J) Group	Mean Difference (I-J)	Std. Error	Sig.	95% Confidence Interval	
					Lower Bound	Upper Bound
A	B	−0.139	0.069	0.149	−0.311	0.032
B	C	−0.251	0.069	0.002	−0.423	−0.079
C	A	0.391	0.069	0.000	0.219	0.562

#### CR5 dimension towards the students’ informatics learning outcomes

For the CR5 dimension, Levene’s homoscedasticity assumption is fulfilled, F(2,57) = 0.277, p. > 0.05, therefore, multiple comparisons have been made by Sidak. ANOVA revealed that the proposed model was not significant in the inter-group variable F(2, 57) = 0.415, p. > 0.05, with a small effect size (*η*^2^ = 0.013).

Table 8 shows that multiple comparisons determined that there were no significant differences in CR5 between group “A” and group “B” (p. = 0.935), group “C” and group “A” (p. = 0.748) and group “B” and group “C” (p. = 0.975). In regard to the CR5, the analysis revealed no significant differences in knowledge acquisition and enhancing informatics learning outcomes between groups. Distance school education or extra-curriculum out-of-school distance informatics courses do not have any significant influence on CR5 outcomes. The students in primary school are not familiar with the major security elements that should be addressed include prevention of unauthorized access (confidentiality), prevention of unauthorized alterations or loss to data (integrity), and prevention of compromises to availability of data to authorized individuals. Moreover, the rare attempts to introduce cybersecurity at out-of-school courses collapses immediately due to a lack of students’ self-awareness. According to (NASK, 2019b), 94% of parents claimed they protection of safety and privacy on the Internet is expected from the owners and administrators of websites, and the Polish government. With respect to teachers, practical hands-on lessons in cybersecurity, must start at primary school and target a diverse range of students. Teachers’ and parents’ lack of awareness and/or digital competencies, may contribute to the critical shortage of students’ cybersecurity skills in the nearest future.

**Table 8:** Multiple comparisons of CR5 towards the students’ informatics learning outcomes

(I) Group	(J) Group	Mean Difference (I-J)	Std. Error	Sig.	95% Confidence Interval	
					Lower Bound	Upper Bound
A	B	−0.029	0.054	0.935	−0.163	0.105
B	C	−0.020	0.054	0.975	−0.154	0.113
C	A	0.049	0.054	0.748	−0.084	0.183

## Discussion

The pandemic forces a large number of schools will remain closed, and distance informatics learning will substitute for the real thing (Onyema et al., 2020). It’s been an extraordinary — and extraordinarily fast — transition, affecting everyone from the youngest children entering school right up to young adults in universities. Pencils, paper and crayons? No, this year’s most important back-to-school supply is a laptop and tablet.

The obtained results, posed in the first research question, justify the study (GUS, 2020b; OECD, 2021b) that declared that over 90% of Polish households have an Internet connection at home, which is above the world average. According to (ITU, 2020) over 57% of households around the world and over 85% of households in Europe today have Internet access at home. Moreover, the obtained results confirm the study (ITU, 2021) that demonstrates over 47% of households around the world and over 77% of households in Europe have access to a computer/laptop. Poland has not been a front-runner in Europe when it comes to equipping schools and furnishing households with ICTs (OECD, 2020). However, the pandemic fostered the government and municipalities to provide targeted policies towards the ICT investment, integration of ICT in teaching and learning, and more specific challenges faced by schools and households. In reaction to the impact of COVID-19 on education, the Zdalna szkoła project (Zdalna szkoła plus, 2020) was founded to provide households in need with ICT devices (51.000 laptops or 120.000 tablets) that will allow students to study from home. Another project (OSE, 2020) delivered more than 12 thousand laptops to schools and households to support crisis-prompted distance education. Of course, there is a small percentage of households that lack computers/laptops, or proper Internet connection to handle the distance education classes. The authors did not consider the situations where the households have access to ICT devices, but the number of ICT devices is less than the number of users. Moreover, the access to the Internet question did not emphasize the quality of the Internet connection. Due to the uneven access to fixed broadband Internet, something, mobile Internet is a more common and more affordable connection option in Polish households. Since these factors were not measured in this study, the following statement should be digested with caution as an alternative statement may hold. Generally, during the COVID-19 pandemic, Polish primary school students could fully benefit from new technologies that include access to the Internet, mobile phones and laptops and other basic ICT devices. Moreover, nowadays parents and teachers struggle with the more sophisticated issues, namely how to coordinate students to strike a balance between using screens, self-efficiency, mental health and crisis, coping with stress, digital fatigue, cyberbullying, online abuse and numerous other consequences and e-threats of crisis-prompted distance education (Tomczyk & Walker, 2021; Lazarinis et al., 2020; Rothe et al., 2021; Sinko et al., 2021; Zuo et al., 2021).

Nowadays in the fast-changing pandemic digital world, technology-led learning is becoming the norm and students may be using ICTs before they can read and write. Students may use computers during distance education, mobile phones to keep in contact with friends and tablets to do schoolwork in the evening that can add up to many hours over the course of the day. However, despite its potential influence on teaching and learning, the mere presence of ICTs does not necessarily lead to student progress (Li & Ma, 2010). Digital literacy is not limited to hardware, software, or digital media (i.e., it is not necessarily linked to the use of laptops, tablets, smartphones or apps) but can be characterized by attitudes and practices whose roots go back a long way to digital culture of educational systems (Backes et al., 2021). Teachers, parents and students must also be encouraged and supported in using ICTs in instructional contexts (König et al., 2020). Therefore, far-reaching added value, for example, in terms of increased ICTs’ usability and accessibility among students, may not yet be guaranteed in terms of their competencies in informatics-related assignments (Sanders & Scanlon, 2021). Therefore, the authors have posed the second research question where they try to understand if the accessibility and the availability of ICTs could increase students’ informatics learning outcomes in out-of-school primary education settings during the crisis-prompted distance education.

The students’ learning outcomes were grouped into five basic criteria/dimensions (CR1-CR5). The revealed results indicate the significant differences (CR1-CR5) in students’ learning outcomes towards the informatics with and without out-of-school fostering activities. Moreover, the presence of school distance informatics education at the beginning of the pandemic had a significant positive impact on primary students’ outcomes in all dimensions (CR1-CR4), except CR5. According to the new Core Curriculum, the last CR5 dimension corresponds to students’ compliance with the law and safety rules (digital safety, cybersecurity, privacy, etc.). In Poland, a lot of extracurricular courses address programming, creativity, algorithmic, problem-based and game-based learning in robotics, electronics and mechatronics courses, while cybersecurity gets no appropriate attention. Moreover, school informatics lessons reveal that neither digital safety nor cybersecurity does not fall into the focus of teachers. Ethics and privacy get briefly mentioned by some teachers, not enough to successfully face the cybersecurity crisis that is coming our way (Informatics Europe & ACM Europe, 2017). Moreover, the parents’ level of ICT literacy and digital preparedness in conjunction with their availability and capacity to assist were not always perceived as satisfactory and could contribute to increasing inequalities in in students’ CR5 competencies.

According to (WEF, 2020) “while many technologies have emerged as potential solutions to global education gaps, technology use is not an end in itself, but rather can serve as a tool to enable new approaches”. Moreover, “without consensus around a normative vision for education in the new economy and society, fundamental innovation in primary and secondary school content and delivery has remained limited” (Azorín, 2020). Though COVID-19 has had a severe impact on normal educational progress, schools should take this unforeseen opportunity to detect deficiencies and speed up reform of online distance informatics education through innovative course content, state-of-the-art technology and efficient management. Poland should turn this emergency into an occasion to further promote international collaboration and share experiences, knowledge and resources to build a global school online distance informatics education network.

## Conclusions

The present study is addressed to the Polish primary school informatics education in pandemic era. It consists of two interrelated research questions. The first research question was addressed to students and was dedicated to furnishing them with ICT devices for distance informatics education. It reveals that the vast majority of Polish primary school students are fully supplied by ICT devices for distance informatics learning, which shows a significant increase over the past decade (European Schoolnet, 2012). The seconds research question was addressed to the experts, reveals that wide accessibility and the availability of ICTs increase students’ informatics learning outcomes in out-of-school primary education settings. Moreover, it shows the significant differences in students’ learning outcomes towards the informatics education in distance learning mode with and without additional out-of-school accelerating activities. Our findings are of particular interest in light of possible crisis-prompted distance education in future but can also serve to inform government institutions and policymakers seeking to develop effective concepts for successful distance learning.

Several areas of interest could be addressed in future research. A longitudinal design would allow for insights into changes in students’ perceived informatics outcomes by means of self-regulated learning, providing further information about the underlying mechanisms that influence distance learning success in primary school settings. The second research question is focused on tutors’ objective observations towards the students’ informatics outcomes. Future research could aim to incorporate other measures of students’ learning success (e.g., grades, time per task). Finally, the impact of other factors as parents’ support, well-being, integration and other economic and social factors should be investigated in future studies on primary Polish primary informatics education in crisis-prompted distance learning mode.

## Figures and Tables

**Fig. 6:**
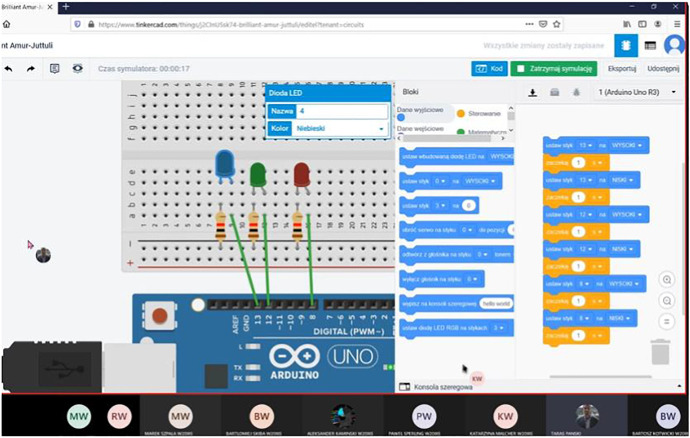
The implementation of solutions in the distance form of extracurricular primary school education. Each student should create a program using multiple LEDs (red, green, yellow) for the correct traffic lights simulation with and without variables

**Fig. 7:**
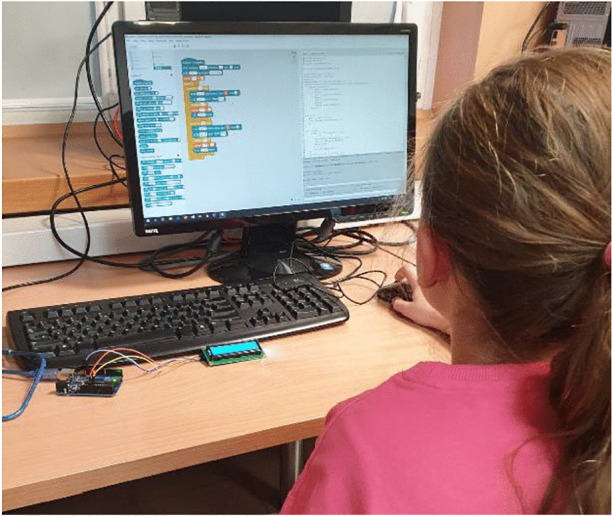
The implementation of solutions in the traditional form of extracurricular primary school education. Each student should create a program that will display the temperature and humidity on the LCD display (Panskyi & Rowińska, 2021)

## Data Availability

Derived data supporting the findings of this study are available from the corresponding author on request.

## Appendix

Survey (sample questions) of students learning outcomes towards informatics issues during the distance out-of-school courses. Questions were created according to the requirements of the new Core Curriculum for informatics in primary schools and were directed to experts. Figures 6 and 7 illustrate the informatics activities in distance vs traditional learning modes during the electronics extracurricular course

**Table:**

1.	Does the student understand the problem?
2.	Is the student able to analyze the problem?
3.	Did the student found a solution to the presented problem?
4.	Can the student transfer his/her concept of solving a problem into the source code?
5.	Can the student program ICT devices?
6.	Is the student able to check the code correctness?
7.	Can the student correct errors(bugs) in the code?
8.	Is the student able to synchronize electronic systems with ICT devices (laptop or computer)?
9.	Can the student analyse different architectures of electronic devices?
10.	Can the student take part in brainstorming activities?
11.	Can the student present his work and solutions?
12.	Does the student know the rules of using the Internet resources?
13.	Does the student know the basics of copyright law?
